# Wer Wissen hat, hat Autorität? Einführung in den Themenschwerpunkt „Wissen in der Ungewissheit“

**DOI:** 10.1007/s12054-021-00450-3

**Published:** 2022-01-05

**Authors:** Jessica Prigge, Lukas Schildknecht

**Affiliations:** 1 Technische Universität Dortmund, Dortmund, Deutschland; 2 Kassel, Deutschland

**Keywords:** Moderne, Soziale Arbeit, Sozialpädagogik, Wissen, Information, Ungewissheit

## Abstract

Wissen ist in der modernen Sozialpädagogik in besonderem Maße mit Ungewissheiten konfrontiert, die jenseits bloßer Informationen liegen. In diesem den Themenschwerpunkt einleitenden Beitrag werden Herausforderungen auch am Beispiel der Pandemie veranschaulicht um dafür zu plädieren, dass eine steigende Komplexität nicht dazu führen muss und darf, Wissen in der Sozialpädagogik als ausschließlich relativ und willkürlich anzusehen. Auch anhand der Beiträge wird in verschiedenen Handlungsfeldern und -logiken das Besondere im Verhältnis von Wissen und Nicht-Wissen aufgezeigt, so dass vielmehr eine reflexive Arbeit mit den jeweiligen Wissensformen notwendig wird.

**Figure:**
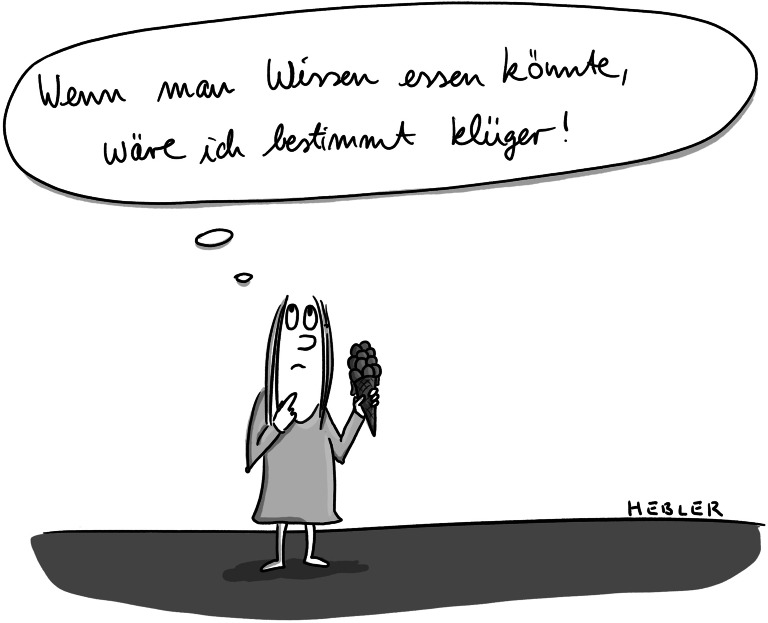


In der Moderne wird Wissen und wissenschaftliches Wissen in der Bearbeitung gesellschaftlicher Probleme herangezogen. Gleichzeitig wird es problematisch, weil es den Bereich des Ungewissen vergrößert. Für Professionelle in der Sozialpädagogik ergeben sich dadurch besondere Herausforderungen an ihr Wissen.

Max Weber schrieb, „das Wissen davon oder den Glauben daran, (…) daß es (.) prinzipiell keine geheimnisvollen unberechenbaren Mächte gebe, die da hineinspielen, daß man vielmehr alle Dinge – im Prinzip – durch Berechnen beherrschen könne. Das (.) bedeutet: die Entzauberung der Welt. Nicht mehr (…) muss man zu magischen Mitteln greifen, um die Geister zu beherrschen oder zu erbitten. Sondern technische Mittel und Berechnung leisten das.“ (Weber 2021 [1919], S. 593).

Wenn es keine göttliche Ordnung mehr gibt oder naturgegebene Verbindlichkeiten, welche bestimmen, wie Menschen in Gemeinschaften werden, so stellt sich das Problem der Gestaltung der Sozialisation in der Moderne immer wieder aufs Neue – als politisches und pädagogisches (vgl. Lütke-Harmann 2016). Die sozialen Berufe sind nun in das Dilemma eingebunden. Wenn es keinen Fatalismus mehr gibt oder keine Niedertracht eines Tyrannen, den man für die Ungleichheit der Menschen verantwortlich machen kann, dann gilt es, Maßnahmen zu ergreifen diese Verhältnisse abzufedern und sich an diesen mit anderen abzuarbeiten (vgl. Mollenhauer 1959). Die französischen und haitianischen Revolutionen geben zwar die Werte vor – Freiheit, Gleichheit und Solidarität – doch lassen sie den Weg dahin offen.

Die im Sozialwesen arbeitenden Professionellen finden sich in einer Situation wieder, in die verschiedene Anforderungen an ihr Wissen herandrängen. Zum einen müssen sie institutionelle Abläufe und juristische Grundlagen kennen, andererseits ist es insbesondere die ständige Reflexivität von spezifischen Situationen und besonderen Fällen, auf die der_die Sozialarbeiter_in angewiesen ist (vgl. Thole et al. 2016).

Dabei ist Nichtwissen in hohem Maße ein Einflussfaktor auf eben jene Reflexionsprozesse. Nichtwissen muss streng unterschieden werden von Uninformiertheit – ein Defizit an Informationen lässt sich aufholen (vgl. Gamm 2017). Nichtwissen ist viel mehr dann gegeben, wenn die Unbestimmtheit der Zukunft in die Handlungsabsichten der Sozialpädagog_innen einbricht; trotz allen möglichen Wohlwollens kann das gewünschte Ergebnis ausbleiben. Beratung kann nicht fruchten, Pläne von Unvorhersehbarem durchkreuzt werden und die Unterstützung eine Adressat_in überfordern. Keine Erfahrungswerte und kein angeeigneter Stoff können Professionelle vor diesen – an Gelingensbedingungen gemessenen – Misserfolgen schützen.

Max Weber beschreibt die Entzauberung der Welt durch den Prozess der Rationalisierung, den „Glauben“ an der Berechenbarkeit aller „Dinge“. Ausläufer eines solch technisch-instrumentellen Denkens in der Sozialpädagogik finden sich dann beispielsweise in dem Wunsch, das sozialpädagogische Handeln ausschließlich an dokumentierbaren Wirkungen auszurichten (Winkler 2018, S. 1358). Die Dimension des Sozialen lediglich unter technischen Regeln kontrollieren zu wollen, vergisst oder unterdrückt sogar eben den Bereich des Nicht-Wissens, der Kontingenz in Verstehens- und Verständigungsprozessen in menschlichen Beziehungen. Sowohl die Individualisierung von Lebensläufen unter steigenden Handlungsspieleräumen und folglich erhöhtem Entscheidungsdruck der einzelnen Menschen (vgl. Rauschenbach 1994) als auch die Akzentverschiebung sozialstaatlicher Absicherungssysteme hin zu persönlicher Verantwortungsübernahme (vgl. Kessl 2005) vergrößern diesen Bereich des Nichtwissens. Und wird der Blick auf das Feld der professionellen Prävention gerichtet, scheint sich Ungewissheit zu potenzieren, wie Friederike Schmidt in ihrem Beitrag „*Prävention und Ungewissheiten des Aufwachsens von Kindern*“ am Beispiel der Ernährungsprävention (in diesem Schwerpunkt) verdeutlicht. Die Frage nach deren Erfolg kann selbst auf Basis fundierten Wissens über das Problem, was vermieden werden soll, nur schwerlich eindeutig beantwortet werden anhand eines nicht eintretenden Ereignisses, welches weit in der Zukunft liegt. Wie diese Ungewissheit verdeckt wird in den untersuchten Präventionsstrategien, problematisiert sie nicht nur, sondern stellt darin auch Potenziale der Weiterentwicklung präventiver Handlungsstrategien heraus.

## Wissen … am Beispiel der Pandemie

Die Pandemie rund um Covid-19 hat den Umgang mit Wissen und Ungewissheit auch in den sozialen Berufen grundlegend erschwert. In der Pandemie zeigte sich ein enormer Bedeutungsgewinn wissenschaftlicher Erkenntnisse sowohl in der Politik als auch im Alltag der Menschen wie auch in sozialpädagogischen Handlungsfeldern. Zur Eindämmung exponentiellen Infektionsgeschehens galt zunächst eine sofortige Kontaktbeschränkung als hoch relevant. In diesem naturwissenschaftlich geprägten Wissen fehlte es jedoch an sozial- wie auch erziehungswissenschaftlichen Erkenntnissen. So konnten Fragen der Profession Sozialer Arbeit, die auf direkte Beziehungen mit Menschen angewiesen ist, zunächst lediglich pragmatisch bearbeitet werden, z. B. schlicht welche sozialarbeiterischen Tätigkeiten sich durch digitale Technik in das Homeoffice verlagern lassen oder komplexer bei der Frage nach der Systemrelevanz von Kindertageseinrichtungen und ihrer zeitweisen Schließung. Während zu Beginn der Pandemie hierin durchaus das Nach-Hinten-Stellen erziehungs- und sozialwissenschaftlichen Wissens aufgrund des vorläufigen Handlungsdrucks nachvollziehbar sein könnte, irritiert umso stärker, dass die Aufgabe der Betreuung schließlich in der Öffentlichkeit zu kritischen Debatten führte, kaum Diskurse wurden dem Bildungsauftrag der Kindertageseinrichtungen unter Pandemiebedingungen gewidmet (vgl. Prigge et al. 2021). Zur allgemeinen Verunsicherung durch das gesundheitliche Risiko sowie eine ökonomische Unsicherheit waren Professionelle zunehmend weiteren Herausforderungen an ihr Wissen ausgesetzt. Sich teilweise innerhalb weniger Tage ändernde Regelwerke von Politik oder den Trägern von Einrichtungen erforderten unfassbare Anstrengungen informationstechnisch nicht hinterher zu hinken und logistische Improvisationskünste diese umzusetzen. Über Jahre tradierte, eingespielte Tagesabläufe und ihre (Wissens‑)Routinen standen komplett zur Disposition.

Zu diesen Ungewissheiten ist noch nicht der erhöhte Unterstützungs- und Beratungsbedarf angesprochen, der aus den Ängsten der Pandemie erwächst. Die Sorge vor Ansteckungen und schweren Verläufen sind dabei ebenso relevant wie Befürchtungen, ob der wirtschaftlichen Engpässe in vielen Berufszweigen oder das Wegfallen von Geselligkeiten. Professionelle stehen also ständig vor Fragen, auf die Wissen verschiedene Antworten gibt. Darunter zählt auch die Frage, wann die Pandemie zu Ende ist. Darüber hinaus gab es die schwierigen Fragen, die die Tagespolitik tangierten. Wie erkläre ich jungen Menschen beispielsweise die Schließung ihres Jugendhauses und der Kindertageseinrichtung ihrer kleinen Geschwister, während ihre Eltern weiter in Großraumbüros und Werkstätten arbeiteten? Katharina Vogel setzt sich in ihrem Beitrag „*Wissen, nicht Wahrheit*“ (in diesem Heft) systematisch mit verschiedenen pädagogischen Wissensformen auseinander und greift auf das Beispiel der Pandemie zurück. Denn u. a. daran gelingt es ihr das Verhältnis von wissenschaftlichem und pädagogischen Wissen zu beleuchten, ohne dass verschiedene „Sinnwelten“ in Beliebigkeit nebeneinanderstehende subjektive Wahrheiten erzeugen.

## … und Information

In der Pandemie ist besonders deutlich geworden, wie sehr modernes Wissenschaftswissen in alle heutigen Lebensbereiche vordringt. Heutige Gesellschaften lassen sich folglich als Wissensgesellschaften (Stehr 2001, S. 10) beschreiben. Wissen scheint in ihnen im Überfluss vorhanden. Über die Digitalisierung könnte man meinen, Unmengen dieses Gutes seien lediglich einen Touch oder Mausklick entfernt. Dabei verlieren tradierte Formen des Wissens und Wissenserwerbs zunehmend an Bedeutung, ihre Legitimität wird fragil. Die Schattenseiten des Digitalen sind dabei nicht zu leugnen. Diskriminierende Äußerungen lassen sich anonym online scheinbar ohne Hemmschwellen verbreiten – Misogynie, Rassismus, Homo- und Transphobie ebenso wie Antisemitismus überschwemmen die Foren und Chatfunktionen. Doch was im Digitalen einen spielerischen Charakter hat, hat ganz reale leibliche Folgen. Der seelische Schmerz der Angegriffenen ist echt. Dass sich verbale Entgleisungen in mörderische Gewalt steigern können, ist traurige Realität.

Das sich eilig verbreitende Medium im Digitalen ist die Information (vgl. Vogl 2021). Eine Information ist ein Gehalt, der, anders als wissenschaftliches oder solides journalistisches Wissen, keiner Überprüfung bedarf. Die Verbreitung von falschen Fakten (teils auf Fehlinterpretation, teils aus politisch motivierter Verfälschung) beschleunigt sich so und führt zu eigenwilligen Dynamiken (vgl. Butter 2018). Bar jeden Gehalts glauben Menschen zu wissen, dass eine jüdische Weltverschwörung tödliche Impfungen einführt, dass Europa ein Bevölkerungsaustausch bevorstehe oder dass ein Staatsfeminismus eine zweite DDR einführen wolle. „Wer Wissen hat, hat Autorität. Er [oder sie] kann die anderen belehren“ (Luhmann 1992, S. 149) – in Anlehnung an diese Beobachtung von Niklas Luhmann ließe sich folglich formulieren: Wer Autorität hat, hat Informationen, die er_sie auch oder gerade in einer Wissensgesellschaft verbreiten kann. Es kommt demnach nicht unbedingt auf überprüfbares Wissen an.

Sozialpädagog_innen sind dann in einem Alltag mit Menschen konfrontiert, die diesen Informationen Bedeutungen beimessen. Sie stehen etwas altmodisch dar, wenn sie diese Aussagen mit Wissen korrigieren wollen oder dahinterliegende, menschenverachtende Ideologien entlarven. Doch solange die Verantwortung für die Flut an Desinformation und Hass niemand übernimmt, können die profitorientierten Plattformen doch auf die Schreibenden verweisen und diese dann auf ihre Meinungsfreiheit, Spekulation oder Ironie (vgl. Vogl 2021), bleibt diese bei der Aufklärungsarbeit von Sozialpädagog_innen hängen. Zwei Beiträge in diesem Heft widmen sich dem Problem, wie die Sozialpädagogik mit menschenverachtenden Phänomenen umgehen kann. Melanie Kuhn und Sandra Landhäußer (in diesem Schwerpunkt) setzen am Rassismus an, innerhalb dessen sie die Verwobenheit von Wissen und grundsätzlichen Überzeugungen und Normen aufzeigen. Sie fragen, wie Lehrkräfte mit rassistischen Äußerungen im unterrichtlichen Geschehen an Fachschulen für Sozialpädagogik umgehen können bzw. provozieren mit der Frage „*Alternative Wahrnehmungen der Welt gelten lassen*?“ Eine rassismuskritische Antwort entwickeln sie aus der Analyse von auf Rassismus bezogene Wissensformen, was auch daran erinnert, dessen, worauf sich Wissen bezieht, Bedeutung beizumessen.

Katja Schau und Carmen Figlestahler führen aus, wie „*Ungewissheiten in der Prävention und Distanzierung von islamistischem Extremismus*“ gefasst werden können, sowohl im sozialpädagogischen Handeln allgemein, als auch in der Prävention von Radikalisierung. Besonders beleuchten die beiden Autorinnen aber die Diffusität des Feldes auf verschiedensten Ebenen und zeigen, dass sozialpädagogisches Handeln trotz aller Widrigkeiten möglich ist bzw. dazu beitragen kann, diesen „Diffusitäten“ zu begegnen. Was vielleicht erstaunen mag, sind Ähnlichkeiten, die sich in der Planung und Überprüfung von Präventionshandeln in so unterschiedlichen Bereichen wie der Radikalisierungsprävention sowie in der Ernährungsprävention bei jungen Kindern aus dem Beitrag von Friederike Schmidt zeigen. Damit verweisen beide Beiträge auf das Allgemeine in der Prävention als Aspekte eines dominanten Risikodiskurses, deren affirmativer Gehalt hier kritisch reflektierbar wird (Winkler 2018, 1359).

## … in der Ungewissheit

Luhmann weist darauf hin, wie problematisch wissenschaftliches Wissen ist, wenn er anmerkt, dass eine Orientierung daran vor allem Unsicherheiten vergrößert (Luhmann 1992). Oder anders, mit ihren Lösungen für bekannte Probleme schafft Wissenschaft ungewisse neue Problemlagen. Die Vermehrung von Wissen „darf nicht als Ausschaltung von Risiken, Zufall, Willkür (…) missverstanden werden“ (Stehr 2001, S. 7). So können dank moderner Medizin heute „symptomlose Infizierte“ erkannt werden, von deren Existenz vorher nichts gewusst wurde, die nun eine ganze Reihe neuer Probleme im Umgang mit dem Infektionsgeschehen aufwerfen (Impfgegner*innen hingegen sind kein neues Phänomen, eine Lösung schien aber auch in der Vergangenheit nicht gefunden worden zu sein, vgl. Leven 2021). Heißt dies alles, dass Wissen obsolet ist? Mitnichten, es zeigt (steigende) Komplexitäten auf, nicht nur für naturwissenschaftliche Themenbereiche. Um der Vielfalt von Wissen im Sozialen gerecht zu werden, ist es zentral, Wissen nicht nur kognitiv zu fassen, sondern, wie in den Beiträgen deutlich wird, verschiedene Wissensformen in unterschiedlichen Zusammenhängen zu unterscheiden, wobei auch implizite, inkorporierte und performative Formen des Wissens in der Sozialpädagogik nicht zu übersehen sind. Ungewissheit kann unterdessen als konstitutiver Bestandteil sozialpädagogischen Handelns gefasst werden (vgl. Dewe und Otto 2015). Auch bleiben Professionelle auf wissenschaftliches Wissen angewiesen, um diskret und innehaltend Bedingungen, Folgen und Grenzen ihres Handelns (Winkler 2018) auch im Kontext ihrer eigenen Positioniertheit in der Gesellschaft machtkritisch zu befragen (Kessl 2021) und um das Soziale reflektiert gestalten zu können. Die Fragen, die in diesem Schwerpunkt aufgegriffen werden, nehmen daher stärker in den Blick, wie sich Ungewissheiten – nicht Uninformiertheiten – im Verhältnis verschiedener Wissensformen, in der Ernährungsprävention bei Kindern, in der Radikalisierungsprävention und Rassismuskritik in der Verwobenheit von Wissen und Normen, jeweils darstellen und welche Fallstricke in Versuchen zur Reduktion von Ungewissheit liegen können. Sie nehmen sich somit der Relation von Wissen und Ungewissheiten in den jeweiligen Handlungsfeldern und -logiken an. Wissen bleibt so zentraler Bezugspunkt des Schwerpunkts. Zwar nicht als absolute oder definierbare Kategorie, aber als Anstoß zur Reflexion und der Arbeit mit Wissen.
